# Cellular Effects of Curcumin on *Plasmodium falciparum* Include Disruption of Microtubules

**DOI:** 10.1371/journal.pone.0057302

**Published:** 2013-03-07

**Authors:** Rimi Chakrabarti, Parkash S. Rawat, Brian M. Cooke, Ross L. Coppel, Swati Patankar

**Affiliations:** 1 Department of Biosciences and Bioengineering, Indian Institute of Technology Bombay (IITB), Mumbai, India; 2 Department of Microbiology, Monash University, Melbourne, Victoria, Australia; 3 IITB-Monash Research Academy, IIT Bombay, Mumbai, Maharashtra, India; Kenya Medical Research Institute (KEMRI), Kenya

## Abstract

Curcumin has been widely investigated for its myriad cellular effects resulting in reduced proliferation of various eukaryotic cells including cancer cells and the human malaria parasite *Plasmodium falciparum*. Studies with human cancer cell lines HT-29, Caco-2, and MCF-7 suggest that curcumin can bind to tubulin and induce alterations in microtubule structure. Based on this finding, we investigated whether curcumin has any effect on *P. falciparum* microtubules, considering that mammalian and parasite tubulin are 83% identical. IC_50_ of curcumin was found to be 5 µM as compared to 20 µM reported before. Immunofluorescence images of parasites treated with 5 or 20 µM curcumin showed a concentration-dependent effect on parasite microtubules resulting in diffuse staining contrasting with the discrete hemispindles and subpellicular microtubules observed in untreated parasites. The effect on *P. falciparum* microtubules was evident only in the second cycle for both concentrations tested. This diffuse pattern of tubulin fluorescence in curcumin treated parasites was similar to the effect of a microtubule destabilizing drug vinblastine on *P. falciparum*. Molecular docking predicted the binding site of curcumin at the interface of alpha and beta tubulin, similar to another destabilizing drug colchicine. Data from predicted drug binding is supported by results from drug combination assays showing antagonistic interactions between curcumin and colchicine, sharing a similar binding site, and additive/synergistic interactions of curcumin with paclitaxel and vinblastine, having different binding sites. This evidence suggests that cellular effects of curcumin are at least, in part, due to its perturbing effect on *P. falciparum* microtubules. The action of curcumin, both direct and indirect, on *P. falciparum* microtubules is discussed.

## Introduction

Curcumin or diferuloylmethane (from the root of *Curcuma longa*) is a naturally occurring phenolic derivative, experimentally proven to possess anti-malaria activity against various *Plasmodium* species [1]–[3]. Its anti-malaria activities have been partially attributed to pro-oxidant properties and the ability to inhibit histone acetyltransferases [1]. The pro-oxidant activity of curcumin is also believed to be responsible for its role in immunomodulation [4]. Another study noted that curcumin interacts with the sarcoplasmic Ca^+^ ATPase (SERCA) of *P. falciparum* and suggested that this may account for its anti-malaria mechanism [5]. Finally, recombinant *P. falciparum* glyoxalase (GloI) has been found to be inhibited by curcumin *in vitro* [6]. Thus curcumin appears to have several targets in the parasite that singly or collectively may be responsible for its observed antiparasitic action.

Curcumin is also known to have multiple targets in mammalian cells, showing a variety of effects including anti-inflammatory activity [7], anticarcinogenic activity [8] and modulation of angiogenesis [9]. These physiological and therapeutic effects are attributed primarily to its antioxidant activity [10], [11]. In addition to this activity, curcumin is known to target transcription factors *c-jun* and *c-fos* [12], NF-κB [13], cytokines and growth factors IL-1, IL-8 [14], FGF-2 [15], receptors and enzymes COX-2 [16], EGFR [17], iNOS [18] and others.

Another target of curcumin in mammalian cells is tubulin, a protein that is essential for cell division and cellular trafficking. Curcumin has been demonstrated to down-regulate the expression of tubulin genes in HT-29 and Caco-2 colon-cancer cells [19]. Curcumin treatment also leads to the formation of mono-polar mitotic spindles in MCF-7 cancer cell lines which were incapable of chromosomal segregation [20]. In another study it was found that curcumin can bind strongly to tubulin *in vitro*, inhibiting polymer assembly, reducing GTPase activity and inducing tubulin dimer aggregation. Additionally, in cells treated with curcumin, depolymerization of mitotic microtubules during cell division was also seen [21].

Although curcumin has not been analyzed for its anti- microtubule activity in *P. falciparum*, various known microtubule inhibitors have been tested in this parasite for their antiproliferative effects. These include microtubule stabilizing agents - taxoids [22], [23] and various destabilizing agents - colchicum alkaloids [24], [25], vinca alkaloids [26], dinitroanilines [27] and tubulozoles [28], [29]. The effects of microtubule inhibition by these compounds on the erythrocytic cycle of *P. falciparum* have been suggested due to defects in cellular processes that are thought to be dependent on microtubule function: merozoite formation [23], inhibition of invasion of erythrocytes by daughter merozoites [25] and blocking of nuclear division [22]. Inhibition of protein synthesis is also an effect of these compounds [29].

As curcumin is known to have inhibitory effects on both mammalian tubulin and *P. falciparum* growth in culture, we hypothesized that *P. falciparum* growth inhibition induced by curcumin might be attributed in part to its anti-microtubule activity in the parasite. Here we have investigated the effect of different concentrations of curcumin on the organization of *P. falciparum* microtubules in cultured parasites. Results are reported through immunofluorescence studies and predicted binding of curcumin to the parasite α, β -tubulin heterodimer by molecular docking. Different mechanisms including direct and indirect effects on tubulin have been discussed to ascertain probable causes for the observed anti-microtubule effects.

## Materials and Methods

### Parasite culture

The chloroquine sensitive strain of *P. falciparum*, 3D7, was grown in human B+ erythrocytes at 5% hematocrit in complete medium containing RPMI 1640 supplemented with 25 mM HEPES, pH 7.5, 25 mM sodium bicarbonate, 50 mg/liter hypoxanthine, 10% Albumax II and 40 µg/ml gentamicin sulphate as previously described [30]. Cultures were maintained at 37°C in a gas mixture of 5% CO_2_,1% O_2_ and 94% N_2_. Synchronization was performed using 5% D-sorbitol [31].

### Ethics statement

Ethics approval was obtained from the Monash University Human Ethics Committee for using human erythrocytes for culturing malaria parasites. Blood was provided by the Australian Red Cross Blood Bank, Melbourne.

### *In vitro* drug susceptibility assays

100 mM stock solution of curcumin was prepared in 100% dimethyl sulfoxide (DMSO), filter sterilized and stored in aliquots at −20°C. The effect of curcumin on parasite *in vitro* growth was tested using a standard Giemsa staining method with serial dilutions of the drug to final concentrations of 0.5 to 50 µM. Synchronized ring stage parasites (0.5%) with 3% hematocrit were seeded in a 24 well plate and curcumin at various concentrations was added along with media up to 96 hours. DMSO (0.1%) was used as solvent control. In all the test cultures DMSO concentration was kept at 0.1% v/v. Parasitemia was determined up to 96 hours. Slides were also photographed at different time points.

For combination assays, combinations of curcumin with colchicine, paclitaxel and vinblastine were set up in 24 well plates following the Fixed Ratio method [32]. In addition, all the four drugs were individually tested. Negative controls had no drug and solvent controls were set up with 0.1% DMSO (v/v). The plates were incubated at 37°C for 96 hours with periodic change of media (containing appropriate drug concentration) every 24 hours. Parasitemia was determined by light microscopy using Giemsa stained smears. Combination Index [CI_AB_ (ΣFIC_x_)] was calculated as:
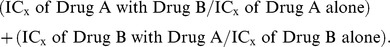



### Hemocompatibility assay for curcumin

Hemolysis was measured as previously described [33] with slight modifications. Blood in 10% CPDA buffer was washed with an equal volume of phosphate buffered saline (PBS) three times. A suspension of erythrocytes (50% hematocrit) was prepared by adding an equal volume of PBS to the washed, packed erythrocytes. To 1.5 ml PBS, 50 µl of cell suspension was added. This sample served as the negative control for hemolysis. 50 µl of cell suspension was also added to 1.5 ml deionized water (hypotonic for erythrocytes), which worked as the positive control for hemolysis. In test samples, 1.5 ml PBS, 50 µl cell suspension and various curcumin concentrations (5 to 100 µM) were added. Spectrophotometer blanks for each test sample were prepared by adding 1.5 ml PBS and various curcumin concentrations (5 to 100 µM) respectively. The experiment was done in duplicates. All the samples were incubated at 37°C for an hour and the reaction was stopped by adding 50 µl of 2.5% glutaraldehyde. The samples were then centrifuged at 1000× g at 4°C for 5 minutes and absorbance of the supernatant was measured at 540 nm. Percent hemolysis was calculated as
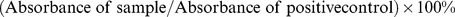



### Pre-treatment assay

Uninfected B^+^ erythrocytes at 3% hematocrit were incubated in complete medium along with 5 µM curcumin and maintained for 48 hours as described. These samples represent the curcumin pre-treated erythrocytes which were subsequently infected with parasite-infected erythrocytes to a final parasitemia of 0.5% (ring-stage) and final hematocrit of 3%. The starting ratio of pre-treated to non pre-treated erythrocytes (uninfected) in the pre-treatment assay samples was 9: 1. These samples were maintained and treated with curcumin, 5 µM for 96 hours. During the same experiment, 0.5% ring-stage parasites were also seeded in erythrocytes (not treated with curcumin) to a final hematocrit of 3% simultaneously. These previously untreated cultures were then treated with curcumin, 5 µM for 96 hours. At each 24 hour time interval parasitemia in various cultures were assessed. All samples were tested in duplicate.

### Curcumin uptake assay

Curcumin uptake assay was performed to assess the concentration of curcumin inside erythrocytes. Experiments were performed with 3 flasks each for first and second cycle of parasite growth. These 3 flasks were - a. Control with no drug b. Treated: with 5 µM curcumin and c. Uninfected erythrocytes: with 5 µM curcumin. Parasitemia in control and treated flasks were kept same at the start of the experiment whereas hematocrit in all the flasks (control, treated and uninfected erythrocytes) was kept at 3%. Uptake assay by fluorimetry was performed as previously described [34]. Fluorescence readings from control flasks (without curcumin) were considered as background and readings from treated and uninfected erythrocytes flasks were normalized with respect to this background reading. A standard curve for curcumin fluorescence was plotted with different concentrations of the drug to correlate fluorescence intensity with curcumin concentration.

### Immunofluorescence

Microtubules in *P. falciparum* in untreated and treated cultures were monitored at various time points and concentrations by confocal microscopy. The primary antibody used for tubulin immunofluorescence (a kind gift from Dr. Angus Bell, Trinity College, Dublin) was raised in rabbit against a *P. falciparum* specific beta tubulin peptide [35]. Acyl Carrier Protein (ACP) antibody specific for *P. falciparum* (a kind gift from Prof. Geoff McFadden, The University of Melbourne) was used for apicoplast immunofluorescence. The immunofluorescence protocol was similar to that reported [36] with a few minor changes. A solution of 4% paraformaldehyde and 0.1% Triton X-100 was used for fixation and permeabilisation. MOWIOL® 4-88 (Calbiochem®) was used as an antifade agent. Images were taken using a Nikon Eclipse 90i microscope.

### Molecular docking

The crystal structure of *P. falciparum* tubulin has not yet been determined, so individual monomers of alpha and beta-tubulin were modeled based on the principle of homology modeling by SWISS-MODEL server [37]. Structures of both the monomers were validated using ANOLEA program in the COLORADO 3D server [38]. The dimeric form of parasite tubulin was constructed by assembling these two monomers using the protein-protein docking server HEX [39].

The crystal structure of curcumin has been published [40]. It has been reported that curcumin, in its solid state, exists as a mixture of its keto and enol tautomeric forms. 3D structures of the diketo (CID969516) and enol (CID11947775) form were downloaded from PUBCHEM. The PUBCHEM structures were in SDF format which were converted to PDB using the NCI Online SMILES Translator (http://cactus.nci.nih.gov/translate/).

Molecular docking of the protein dimer with curcumin and colchicine was performed using AUTODOCK v4.0 [41]. Clusters were generated with an RMS tolerance of 2 angstrom. The Lamarckian Genetic Algorithm (LGA) was used and 250 GA runs were performed for each docking with 1500000 energy evaluations per run. Docked poses were rendered with PyMOL (The PyMOL Molecular Graphics System, Version 1.2r3pre, Schrödinger, LLC).

## Results

### Curcumin inhibits growth of *P. falciparum* cultures *in vitro*

**Figure 1:**
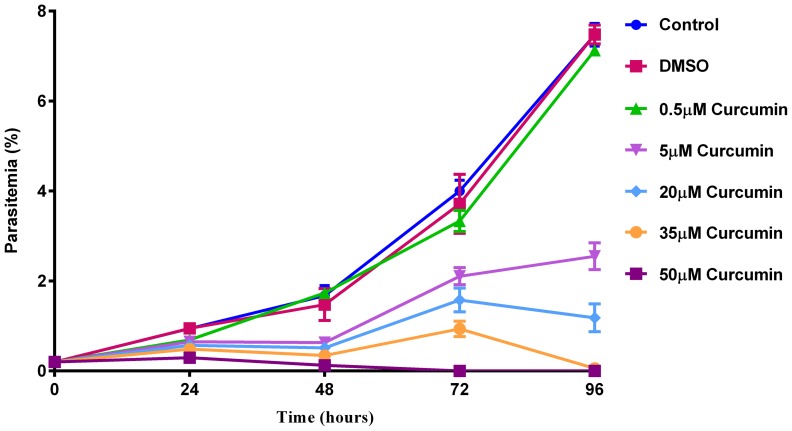
Inhibitory effect of curcumin on *P. falciparum* growth. Control = no drug administered. Solvent control, DMSO = 0.1% (v/v) DMSO. Parasitemia was determined by light microscopy of Giemsa stained blood smears. Error bars represent standard error of the mean (n = 4).

Previous reports have demonstrated the anti-malaria potential of curcumin with IC_50_ values ranging from 5 to 30 µM [1], [2], [42]. In order to determine the working concentration for 3D7 parasites, an *in vitro* susceptibility assay against *P. falciparum* was performed. Figure 1 shows the anti-malaria activity of curcumin. Treatment of *P. falciparum* cultures with curcumin demonstrated a dose dependent antiparasitic effect. The solvent control (0.1% v/v DMSO) showed no adverse effect on parasite viability.

At curcumin concentrations of 35 µM and 50 µM, rapid parasiticidal effect was observed, clearing 70–90% of the cultures within 48 hours (corresponding to one *P. falciparum* life cycle). At 20 µM curcumin concentration, the parasite growth was reduced by 63% compared to control in the first cycle. After the first cycle, the cultures treated with 20 µM curcumin also showed minimal parasitemia (15%). The percent parasitemia in cultures containing 5 µM curcumin showed ∼50% reduced growth in the first cycle. However, unlike results with higher concentrations of curcumin, at this concentration the parasitemia remained almost constant after the first parasite life-cycle; at the 96 hour time point a 10 fold increase in parasitemia (compared to 0 hours) was observed at 5 µM curcumin in contrast to 37 fold increase in controls. Together with compromised growth, cultures treated with 5 µM curcumin also showed loss of synchrony after 48 hours. At 0.5 µM curcumin, parasite growth was indistinguishable from that of the controls (no curcumin/solvent).

We noted a degree of parasiticidal activity at curcumin concentrations of 5 µM. This is of interest since Cui *et. al.* have previously demonstrated that after 5 µM curcumin treatment for 4 hours, levels of reactive oxygen species (ROS) were similar to controls [1]. Thus this concentration offers an opportunity to examine curcumin effects that may be independent of generation of ROS, in particular the effects on microtubule structure.

### Curcumin leads to morphological alterations in the parasite

**Figure 2:**
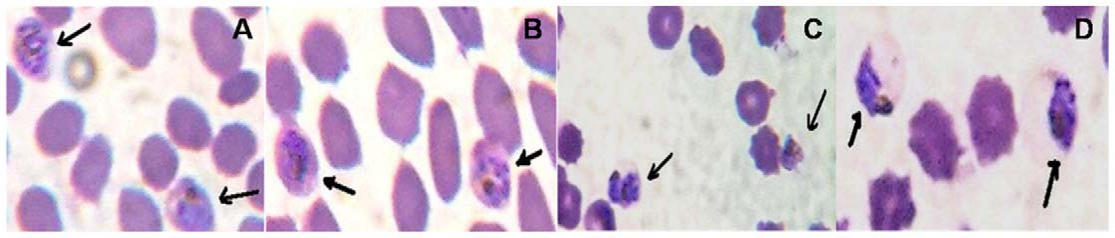
Morphological changes induced in *P. falciparum* by curcumin treatment. Panel A and B represent the morphology of schizonts and late trophozoites in untreated cultures. Panel C and D represent the morphological changes in schizonts and late trophozoites in cultures treated for 96 hours with 5 µM curcumin.

Previously, treatment with microtubule inhibitors has been shown to lead to abnormal morphology of the parasites *in vitro* [22], [23]. To analyze the gross effects of curcumin on parasite morphology, 5 µM curcumin treated cultures were compared with untreated cultures. Figure 2 shows the morphological alterations in *P. falciparum* cultures treated for 96 hours with 5 µM curcumin. In contrast to untreated cultures, nuclear material of the parasites (at schizont stage), in 5 µM curcumin treated cultures, appeared to be shrunken and aggregated concentrating towards one end of the erythrocyte. In addition to the parasites, the morphology of the uninfected erythrocytes in the curcumin treated cultures also appeared distorted (Figure 2, Panels C and D). This led to two hypotheses; first, that curcumin may be hemolytic or second, that curcumin may be affecting the properties of the erythrocytes required for intracellular parasite growth. We examined each of these possibilities.

### Curcumin is not hemolytic

**Figure 3:**
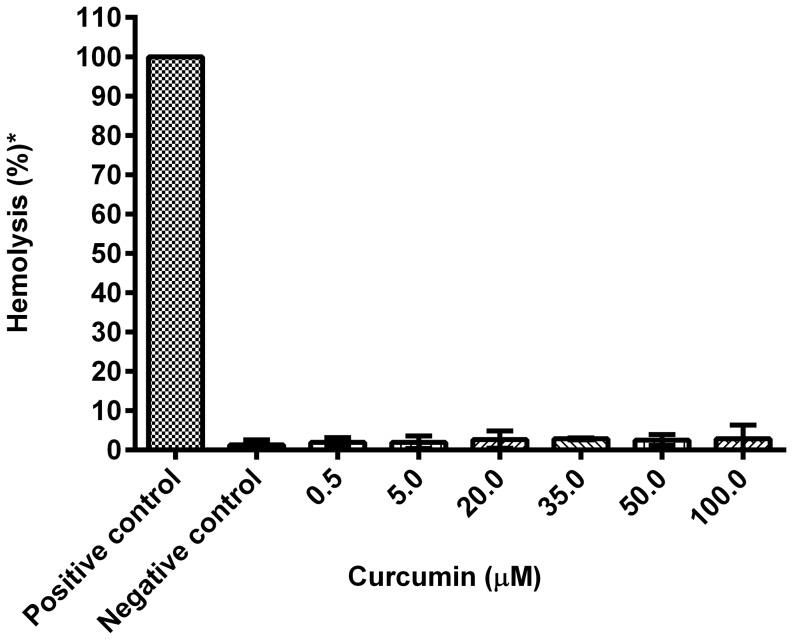
Percentage hemolysis at different concentrations of curcumin. Erythrocytes were resuspended in isotonic PBS (137 mMNaCl, 2.7 mMKCl, 10 m Na_2_HPO_4_, 1.76 mM KH_2_PO_4_, pH 7.0) (positive control), deionised water (negative control), and different concentrations of curcumin. * Hemolysis was measured as a % of positive control. Error bars represent standard error of the mean (n = 6).

The hemolytic potential of curcumin was determined by incubating isolated erythrocytes with different concentrations of curcumin (0.5–100 µM). Compared to the positive control (deionized water, hypotonic), all curcumin concentrations showed negligible hemolytic activity (Figure 3), and all were within the permissible limit of 5% for hemolysis [43]. This suggests that the anti-plasmodial activity of curcumin is not a result of a significant hemolytic effect of the compound.

### Curcumin does not modify erythrocyte properties required for growth of *P. falciparum*

**Figure 4:**
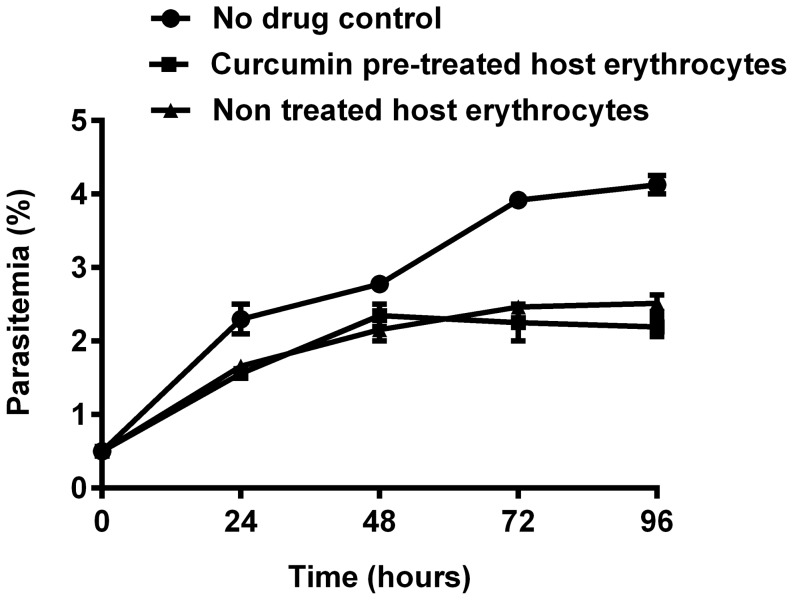
Comparative growth patterns of cultures containing curcumin pre-treated and non pre-treated host erythrocytes. For pre-treatment, erythrocytes were incubated with 5 µM curcumin for 48 h and then mixed with parasite-infected untreated erythrocytes to a final parasitemia of 0.5% and final hematocrit of 3%. In no pre-treatment cultures, erythrocytes were not given any pre-treatment with curcumin. No drug control parasite culture with no drug. Error bars represent standard error of the mean (n = 4).

To address the hypothesis that curcumin alters the properties of host erythrocytes required for adequate growth of the parasites, we pre-incubated erythrocytes with 5 µM curcumin for 48 hours before infecting with *P. falciparum*. If this hypothesis were true, parasite growth should be lower in cultures with pre-treated erythrocytes than in cultures containing non pre-treated erythrocytes. Figure 4 shows the comparative parasite growth patterns in cultures containing 5 µM curcumin pre-treated and untreated erythrocytes. Up to 96 hours, i.e. two parasite life cycles, the parasitemia in both pre-treated and non pre-treated cultures are similar. This suggests that curcumin at 5 µM does not affect properties of the host erythrocytes required for survival and growth of *P. falciparum*. We conclude that the inhibitory effect of curcumin on *P. falciparum* is due to action on parasites leading to reduced parasite growth.

### Effects of curcumin on *P. falciparum* microtubule structures

**Figure 5:**
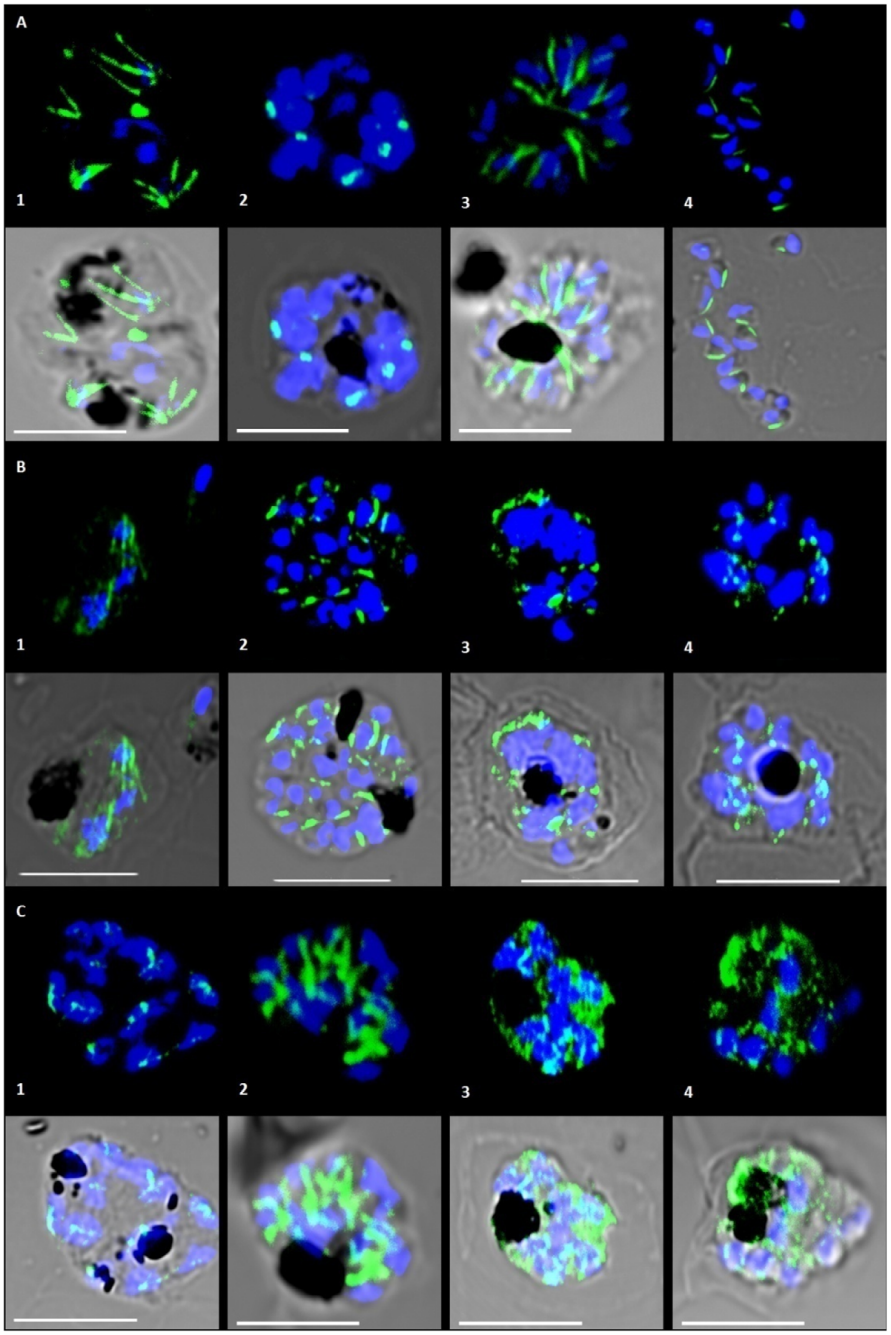
Effect of curcumin on *P. falciparum* microtubular structures. Microtubular structures of *P. falciparum* as observed in different timelines of progression through the mature blood stages. Parasite tubulin is stained with an anti β tubulin antibody and Alexa Fluor 488 and is shown in green. Blue represents nuclear material stained with DAPI. Panel A: Normal parasite microtubules without any drug treatment. Spindle structures were observed in the mid to late trophozoite stages (A1) and distinct MTOCs in the schizonts (A2). The typical subpellicular microtubules were seen in the merozoites (A3 and A4). Panel B: Parasites treated with 5 µM curcumin daily over the course of 96 hours, B1 – 24 hours, B2 – 48 hours, B3- 72 hours, B4 – 96 hours. Panel C: Parasites treated with 20 µM curcumin daily over the course of 96 hours, C1 – 24 hours, C2 – 48 hours, C3- 72 hours, C4 – 96 hours. Spindle structures are conspicuous in their absence in the trophozoites. Towards the later stages tubulin fluorescence pattern becomes diffused compared to the distinct MTOCs and subpellicular microtubules observed in the untreated parasites.

Curcumin has potent anti-microtubule activity in mammalian systems [20], [21] and parasite tubulin shares 83% identity with its mammalian counterpart. This led us to hypothesize that curcumin can also perturb parasite microtubules. To investigate this, immunofluorescence studies were performed using a beta-tubulin antibody specific for *P. falciparum* tubulin. Parasites were treated with 5 and 20 µM curcumin for 96 hours and compared to untreated parasites at similar stages. Figure 5 shows immunofluorescence images of untreated (5A) and curcumin-treated (5 and 20 µM) *P. falciparum* cultures (5B, C).

In untreated cultures, parasite microtubules and the structures formed by them were observed to follow a particular pattern as *P. falciparum* progressed through its different blood stages of rings, trophozoites and schizonts. In the trophozoite stage, at the onset of nuclear division, hemi-spindle structures were seen always associated with the nucleus. In schizonts, subpellicular microtubules were clearly visible. When treated with 5 µM and 20 µM curcumin, changes were observed in microtubular structures, as compared to untreated cultures and no significant effect on parasite microtubule morphology was observed in the first 48 hours (corresponding to the first cycle).The number of trophozoites and schizonts imaged with spindle and subpellicular microtubules in treated cultures were less than controls, but still comparable (Table 1). Major effects were evident after approximately 48 hours when the parasites progressed to the next growth cycle. No definite hemispindle structures could be found throughout the trophozoite stages or any subpellicular microtubules in schizonts (Table 1). Instead, the tubulin immunofluorescence patterns appeared micro-punctate and diffuse in both these stages. Of note here is that although progression through the growth cycle was delayed, there was nuclear division towards the end of the first cycle as well as the second one in the curcumin treated population. No apparent differences were noted from the untreated schizonts in terms of number of nuclei per infected host cell. This late action of curcumin on *P. falciparum* microtubules, occurring in the second cycle, was noted and subsequent experiments were performed to explore potential factors leading to this effect.

**Table 1:** Proportion of total parasite cells observed with spindle and subpellicular microtubules in control and treated parasite population.

	Control	Curcumin (5 µM)	Curcumin (20 µM)
**Spindle microtubules 1st cycle**	87.5%	31.76%	30.76%
**Spindle microtubules 2nd cycle**	90%	0	7.14%
**Subpellicular microtubules 1st cycle**	61.53%	53.57%	26.66%
**Subpellicular microtubules 2nd cycle**	45.83%	0	0

**Figure 6:**
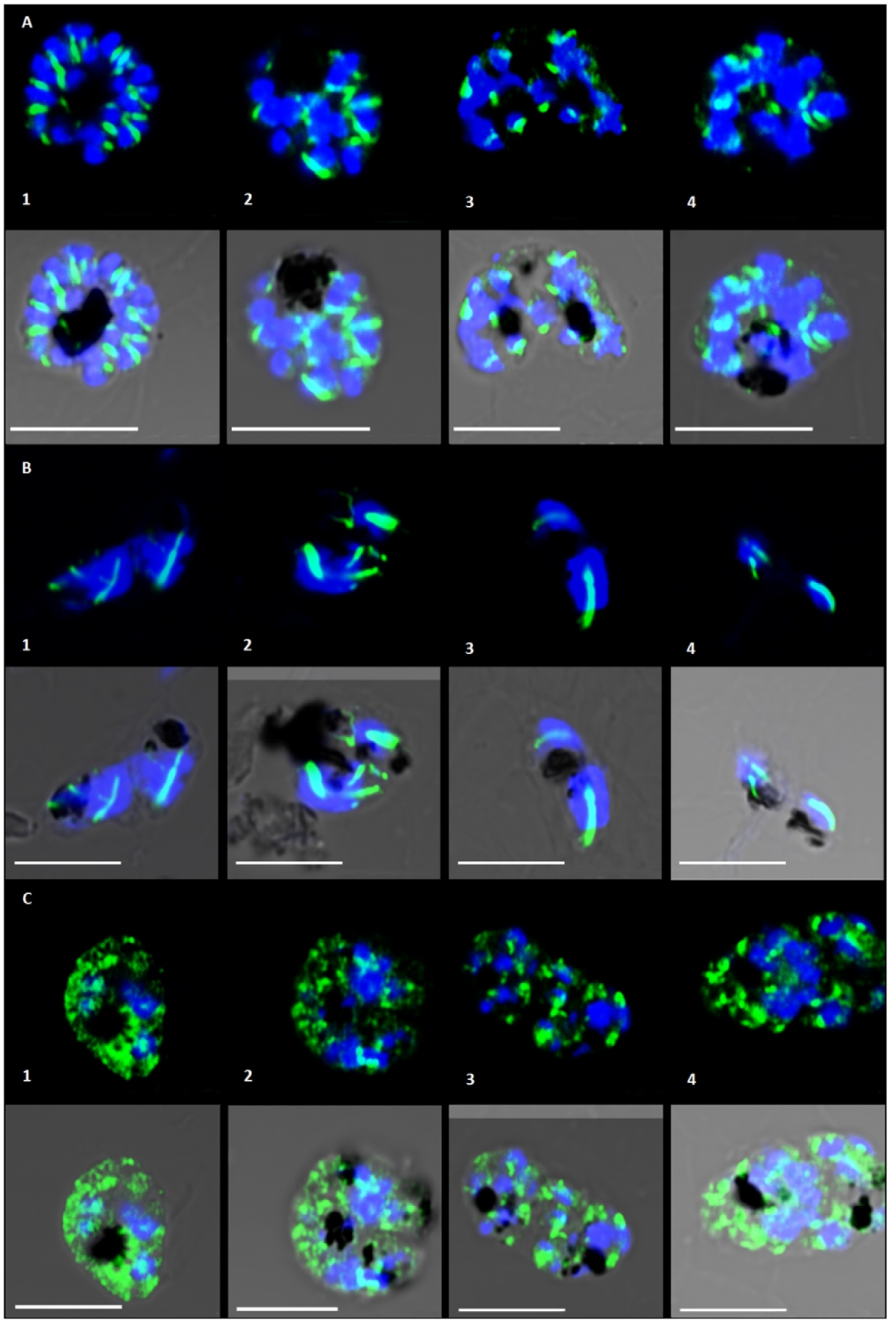
Controls: Treatment of *P. falciparum* with antimitotic drugs. Microtubule stabilizing and destabilizing compounds exert contrasting effects on their target. Panel A shows *P. falciparum* without any drug treatment after 24 hours. Panel B shows parasites 24 hours after treated with 500 nM paclitaxel. Images in panel C represents parasites treated with 100 nM vinblastine, after 24 hours. Molar concentration of the drugs was chosen on the basis of published IC_50_.

The micro-punctate staining and diffuse localization of tubulin with an apparent loss of hemi-spindles suggested that curcumin destabilizes microtubules. To compare these results with those seen for known microtubule destabilizing and stabilizing drugs, we treated cultures with paclitaxel and vinblastine (Figure 6 B, C). Paclitaxel treatment (1 µM for 6 hours) has been shown to result in the appearance of thick rod-like microtubules whereas vinblastine treatment (20 µM for 6 hours) has the converse effect and results in diffuse tubulin staining [35], [44] in *P. falciparum*. We treated cultures for 24 hours with lower concentrations of paclitaxel and vinblastine (500 nM and 100 nM respectively). As expected, paclitaxel-treated parasites exhibited thick rod-like microtubule structures (Figure 6B) and vinblastine treated parasites showed diffuse, micro-punctate staining of tubulin (Figure 6C) resembling the appearance of curcumin treatment cultures. This suggests that cellular effect of curcumin in *P. falciparum* is similar to known destabilizing drugs.

### Intracellular concentration of curcumin is much less than 5 µM

**Figure 7:**
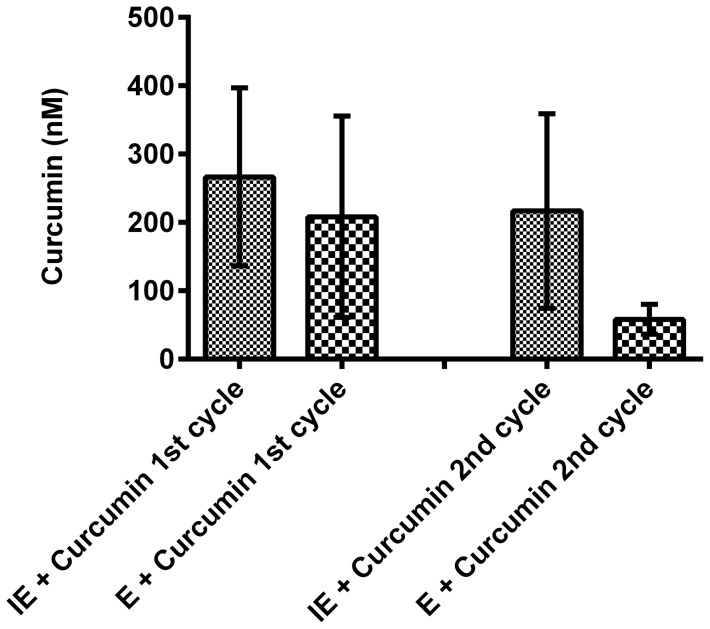
Curcumin uptake by parasitised (IE) and uninfected erythrocytes (E) during first and second cycle of *P. falciparum* growth. Error bars represent standard error of the mean (n = 3).

Immunofluorescence studies showed that the effect of curcumin on *P.falciparum* microtubules was more prominent in second cycle rather in the first cycle. This could be due to several reasons, including low permeability of the compound into the erythrocytes, resulting in lower intracellular drug concentration. To test this possibility, the concentration of curcumin within erythrocytes after the first and second cycle was measured. On average (n = 3), 267 nM of curcumin was found inside the cells of the treated cultures compared to 208 nM curcumin in uninfected erythrocytes, in the first cycle of growth. In the second cycle 217 nM of intracellular curcumin was found in treated cultures compared to 58 nM in uninfected erythrocytes (Figure 7). Note here that 5 µM curcumin was added to the culture for these experiments. Hence, fluorimetric estimation of uptake revealed that a very small fraction (217–267 nM) of this 5 µM curcumin was taken up by the infected erythrocytes. Additionally, no significant differences in intracellular curcumin were observed in the first and second cycles of parasite growth.

### Effects of curcumin on *P. falciparum* apicoplasts

**Figure 8:**
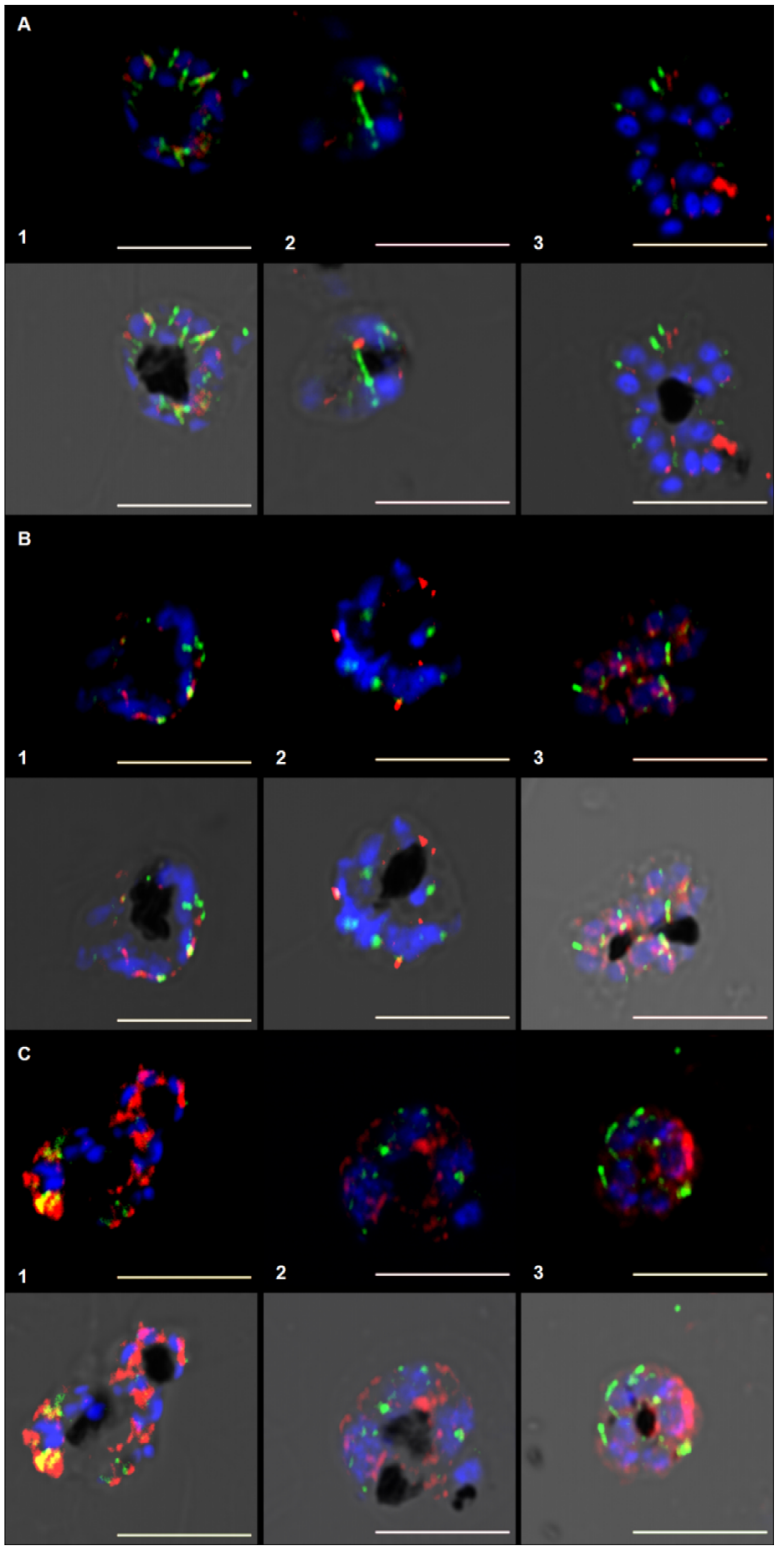
Effect of curcumin on *P. falciparum* apicoplast. Panel A: Untreated parasites at (1) 45 hours (2) 69 hours and (3) 73 hours. Panel B and Panel C represent parasites treated with 5 µM and 20 µM curcumin respectively, at same time points. Tubulin fluorescence is depicted in green, apicoplast fluorescence in red and nucleus in blue.

Late action of curcumin on parasite microtubules, in the second cycle, is concurrent with a large body of evidence reporting apicoplast targeting drugs showing prominent effects in the second cycle after treatment begins [45]–[47]. In eukaryotes, including Apicomplexans, microtubules are known to provide the tracks for segregation of organelles [48]. To address whether apicoplast structure and segregation are affected in curcumin-treated parasites, we visualised apicoplast by immunofluorescence. Apicoplasts in curcumin treated parasites were morphologically different from the untreated controls. Distinct spherical apicoplasts, associated with subpellicular microtubules (Figure 8 A1, A3) and spindle microtubules (Figure 8 A2), were observed in controls and also in the first cycle of 5 µM curcumin treated parasites (Figure 8B1). Parasites treated with 5 µM curcumin at later stage (93 hours) (Figure 8 B3) as well as parasites treated with 20 µM curcumin (Figure 8C1, C2 and C3) showed a diffuse pattern of apicoplast fluorescence, different from the apicoplasts observed in untreated parasites.

### Autodock predicts curcumin binding at the interface of *P. falciparum* tubulin dimer

To provide supporting evidence that curcumin can affect microtubules by destabilizing tubulin polymers, we hypothesised that *in silico* docking of the compound to the α-β heterodimer of tubulin should reveal a binding site close to those of destabilizing drugs. The binding sites of paclitaxel - a stabilizing drug and two destabilizing drugs - vinblastine and colchicine, to mammalian tubulin have been studied in great detail. Paclitaxel and vinblastine has been shown to be bind at the taxol and vinca domain respectively, present in β-tubulin [49] and colchicine to the interface of the tubulin dimer [50].

**Figure 9:**
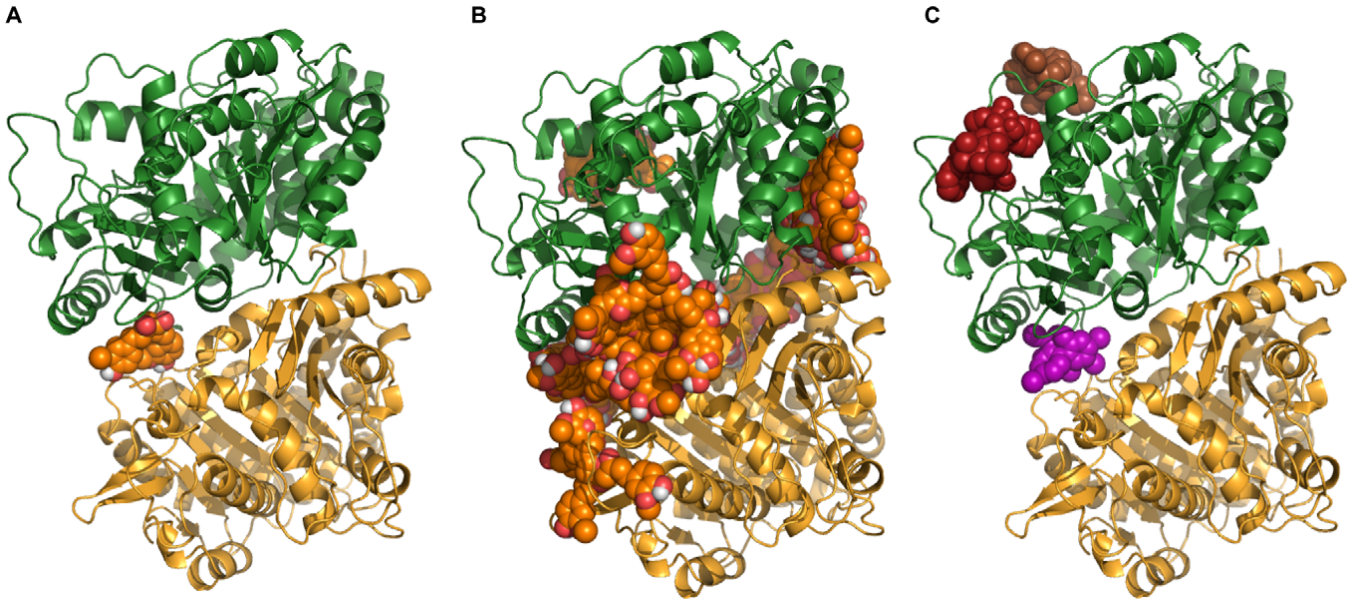
Predictive binding of curcumin, colchicine, paclitaxel and vinblastine to *P. falciparum* tubulin dimer. Figures showing bound poses of curcumin diketo form at the interface of tubulin dimer. *P. falciparum* tubulin is represented as dimer of alpha (yellow) and beta (green) subunit. Panel A shows all the predicted bound poses, mostly at the interface of the dimer. Panel B shows the most probable binding pose according to Autodock (Rank 1) with the curcumin diketo form at the interface of alpha and beta tubulin monomers. Panel C shows binding sites of colchicine (purple), paclitaxel (red) and vinblastine (brown) on parasite tubulin dimer.

Docking studies of curcumin with Autodock resulted in 250 binding poses each for the diketo and enol form of curcumin. The binding of the diketo form is highly site specific. Most of the 250 bound poses are clustered at the interface of the alpha and beta subunits (Figure 9A). The most suitable binding pose as predicted by Autodock, is also at the interface (Figure 9B). This pose was used to compare the interacting residues with those of colchicine.

To this end, colchicine, paclitaxel and vinblastine were also docked with the parasite tubulin dimer to find its binding site. Analysis of interacting amino acid residues revealed the predicted binding site of the diketo form of curcumin to tubulin overlapping the colchicine binding site (Table 2 and Figure 9C). On the other hand, the binding site of the enol form seems to be spread out throughout the dimer (data not shown). Paclitaxel and vinblastine binding sites on *P. falciparum* beta tubulin (Figure 9C) was found to be similar to their binding sites in mammalian tubulin.

**Table 2:** Predicted curcumin and colchicine binding residues in modeled *P. falciparum* tubulin dimer.

A. Curcumin (diketo) interacting residues Rank 1		B. Colchicine interacting residues Rank 1	
Alpha tubulin	Beta tubulin	Alpha Tubulin	Beta Tubulin
Met1	Leu246	Met1	Asn256
Arg2	Asn256	Arg2	Asp327
Lys164	Asp327	Thr130	Met330
Ser165	Pro346	Gly131	Gln334
Asp251	His347	Leu132	His347
Val252	Thr349	Gln133	Thr349
Thr253	Lys350	Asp251	Lys350
	Ser351	Val252	Ser351
		Thr253	

### Curcumin and colchicine show antagonistic interactions whereas combinations of curcumin-paclitaxel and curcumin-vinblastine have additive and synergistic interactions respectively

**Figure 10:**
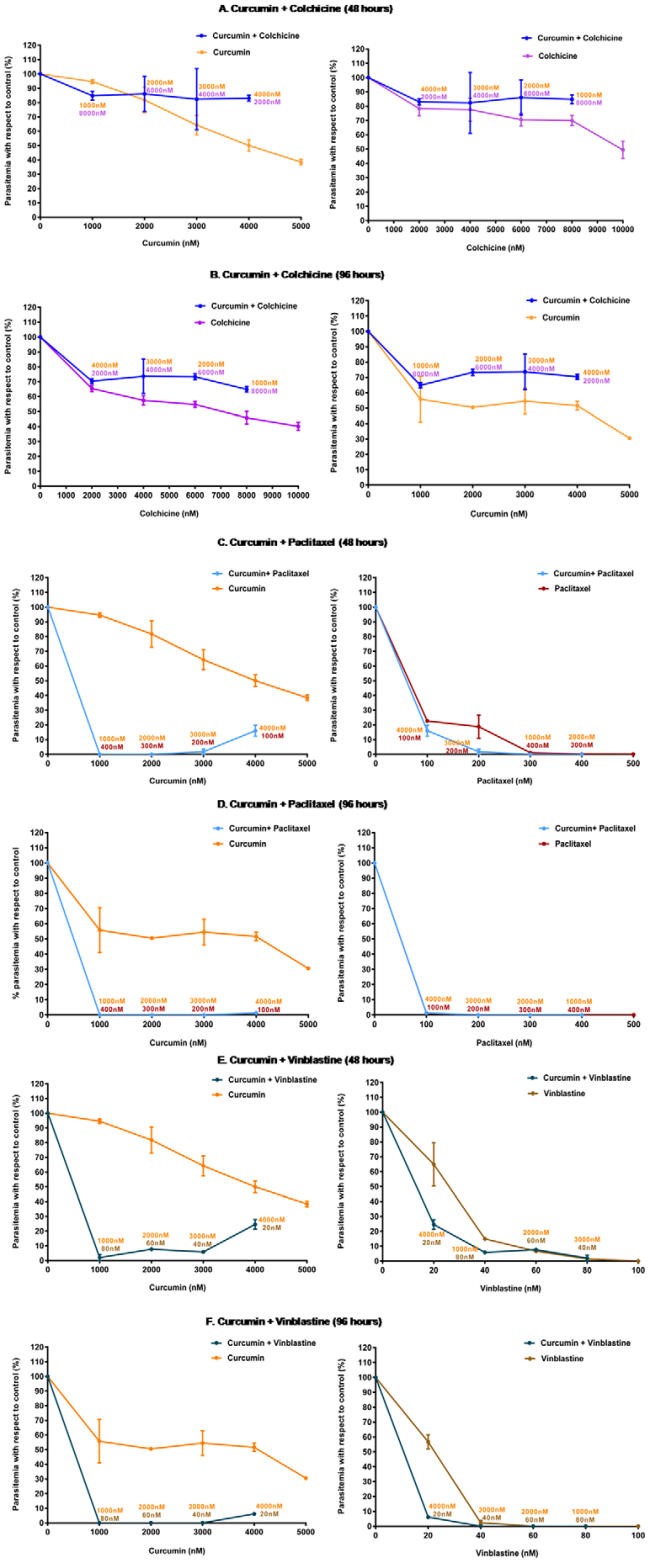
Sensitivity assays with curcumin in combination with colchicine, paclitaxel and vinblastine and all the drugs individually. Panels A and B represent growth patterns of parasites subjected to curcumin and colchine individually and in combination, for 48 hours and for 96 hours respectively. Panels C and D represent same scenario for curcumin and paclitaxel treated parasites while panels E and F represent curcumin and vinblastine treated parasites. Concentrations of individual drugs used in the combinations are included for each data point. Error bars represent standard error of the mean (n = 4).

Molecular modeling of the predicted binding of curcumin to the tubulin heterodimer indicated that colchicine and curcumin have overlapping binding sites while paclitaxel and vinblastine have binding sites distinct from that of curcumin. This prompted us to test the effect of combinations of these anti-microtubule drugs with curcumin, on parasite viability. The growth pattern observed for all the combinations tested, when compared with the growth curves for the effect of individual drugs, showed that when curcumin and colchicine were tested in combination, neither of the two drugs could function at their full potential (Figure 10 A, B). Combination Indices (CI) calculated at two different time points, 6.75 at 48 hours and 2.51 at 96 hours denote antagonistic interaction between curcumin and colchicine. Conversely, curcumin showed an additive interaction in combination with paclitaxel and a synergistic interaction with vinblastine (Table 3).

**Table 3:** Combination indices of curcumin with colchicine, paclitaxel and vinblastine over a course of 96 hours.

	Curcumin			
	CI (ΣFIC15)		CI (ΣFIC90)	
	48 hours	96 hours	48 hours	96 hours
**Colchicine**	6.757	2.513	ND*	ND*
**Paclitaxel**	1.031	1.422	1.645	1.036
**Vinblastine**	0.559	0.919	1.640	0.555

ND* not determined: ΣFIC90 for curcumin-colchicine combination could not be determined because the maximum growth inhibition of treated parasites, even after 96 hours, was 35%.

## Discussion

It has been reported that curcumin has multiple targets in *P. falciparum*, similar to mammalian cells. With a compound like curcumin that acts on many pathways and proteins in a cell, it could be possible that different targets are affected at different concentrations. The 5 µM curcumin concentration was interesting to us for two reasons. First, Cui *et. al.* showed that at 5 µM curcumin levels of ROS, which have been proposed to be responsible for the antiparasitic action of curcumin, were similar to controls [1]. Second, since a fraction of parasites escape the first cycle, the microtubule structures of these parasites can be studied even at 96 hours. So, 5 µM may represent a concentration of curcumin where effects on microtubules and cell numbers are seen with minimal contribution from global effects such as generation of ROS. Our immunofluorescence data suggest that the microtubule-affecting properties of curcumin are evident even at 5 µM.

Apart from elevated ROS levels there was also a chance that curcumin could alter erythrocyte properties that are necessary for parasite survival. After all curcumin is known to affect mature erythrocytes by inducing their suicidal death with involvement of Ca^2+^ influx and formation of ceramide [51]. So when the morphology of erythrocytes seemed to be perturbed at 5 µM, it was possible that the decrease in parasite growth could be due to alteration of erythrocyte properties required for parasite survival. To asses this possibility 5 µM curcumin pre-treated (for 48 h) erythrocytes were inoculated with parasites. Patterns of parasite growth in cultures containing pre-treated erythrocytes and those containing erythrocytes which did not undergo this pre-treatment were similar. This suggests that at a 5 µM curcumin concentration, reduction in parasitemia is unlikely to be a result of alteration of erythrocyte properties required for intracellular growth of *P. falciparum*.

Despite this evidence, unless binding of curcumin to *P. falciparum* microtubules is demonstrated, it is difficult to ascribe the effects of curcumin to a direct interaction with microtubules. Nevertheless, immunofluorescence data shown in this report indicates that curcumin certainly disrupts parasite microtubules. This could also be through secondary routes such as elevated ROS leading to global effects on microtubule stability. However the latter seems unlikely in light of our immunofluorescence assay results with 20 µM curcumin. A previously published report suggested that ROS levels were considerably higher than normal after 4 hours of treatment with 20 µM curcumin [1]. Increased ROS levels are known to lead to increased cytotoxicity. In this context, we note that microtubules of 20 µM curcumin treated parasites looked unperturbed and similar to controls even after 24 hours (Figure 5 panel C1). In the following sections we discuss aspects of curcumin action on *P. falciparum* growth, invoking models of direct action on microtubules as well as indirect effects.

### Predicted binding site of curcumin to tubulin overlaps that of colchicine – a destabilizing drug

The major line of evidence in favor of a direct interaction between curcumin and tubulin is provided by docking studies with the diketo form of curcumin that is predicted to bind to the interface of the tubulin dimer. This binding pattern of the ligand is indicative of its effect on *P. falciparum* microtubules resembling destabilizing drugs, considering that the binding site overlaps with the colchicine domain [50]. Colchicine binds predominantly to beta tubulin at the interface of the dimer. It has been shown to bind tubulin in a biphasic manner and is believed to induce a conformational change on binding [52]. Interestingly, experiments in HeLa and MCF-7 cells have indicated a similar binding site and same mode of action for curcumin [21]. *P. falciparum* tubulin is yet to be purified in sufficient quantity to conduct similar experiments in the parasites. However based on this correlation it seems probable that, like colchicine, curcumin molecules bind to a fraction of the free tubulin, induce a conformational change, thus forming an altered tubulin-curcumin complex. When these defective dimers along with normal dimers are polymerised to the plus end, the microtubules can still grow, but their dynamic properties are compromised.

Drug interaction data from the combination assays supports this line of evidence. Antagonistic interaction between curcumin and colchicine could be due to a) the drugs competing for the same binding site or b) having contradictory effects on the same target. Immunofluorescence assays show curcumin to have a destabilizing effect like colchicine so the competing binding site hypothesis seems most plausible. Of note here is the apparent poor effectiveness of the curcumin-colchicine combinations even after 96 hours (Figure 10B). Had the antiproliferative action of curcumin been due to off target or global effects (e.g. increase in ROS levels), and not due to targeting tubulin, the combination growth curves at 96 hours should have been similar to the individual drugs in case of curcumin-colchicine combinations.

This hypothesis can also be complemented with the results from the controls – the curcumin-vinblastine and curcumin-paclitaxel combination experiments. Vinblastine and paclitaxel are known to bind at the top and one side of beta tubulin respectively (Figure 9C and extensive data from mammalian tubulin structures). These binding sites, distinct from the colchicine as well as curcumin binding site at the interface of the tubulin dimer (Figure 9A–C), could explain the effect of curcumin-paclitaxel and curcumin-vinblastine combinations. In the latter case curcumin and vinblastine, both destabilizing drugs, bind to two separate sites on the beta tubulin and bring about a synergistic effect in reducing the parasitemia. When combined with paclitaxel, curcumin shows an additive effect. This could be partly because paclitaxel and curcumin have two opposing modes of action on tubulin. However, the growth curves for combinations of these two drugs (Figure 10C, D) suggest that paclitaxel is the more potent partner in this combination and seems to be mostly responsible for the observed additive effect.

### Effect of curcumin on microtubules is more pronounced in the second cycle and associated with an altered apicoplast morphology

The microtubule disrupting action of curcumin becomes prominent in the second cycle. This delayed aspect of its action may be attributed to several possible factors. If curcumin acts stage specifically in the mature parasites and considering a tight 100% synchrony is hard to achieve, it might be possible for some parasites to escape the drug action in the first cycle as has been observed previously with vinblastine [26]. Alternatively, it might be due to the fact that rings are less permeable in their uptake of external compounds [35]. However, neither reason explains why some parasites escape the action of the drug even after the second cycle, at lower concentrations.

An alternative explanation could be that curcumin, like the antibacterial agents tetracycline [47], azithromycin, ciprofloxaxin, doxycycline and clindamycin [53], exhibit its effect more prominently in the second cycle. A widely accepted model of the tetracycline mechanism of action in *P. falciparum* is based on loss of apicoplast function [54]. Presently, no evidence has been cited in favor of an interaction between the apicoplast and microtubules in *P. falciparum*. However, in a related apicomplexan parasite, *T. gondii*, division and segregation of apicoplast into daughter cells have been observed to be linked with centrosomes/microtubules [55]. It is possible that the disruption of microtubules seen after curcumin treatment causes a cascading effect which, in addition to disrupting schizogony, also disrupts apicoplast segregation and function.

When treated with curcumin (both 5 µM and 20 µM), parasites showed apicoplast morphology different from the untreated controls at same time points (Figure 8). Based on observations in *T. gondii* [55], tubular, undivided apicoplasts might have been expected in the mature stages resulting from a segregation defect. Instead diffuse apicoplast fluorescence is observed in the mature stages of treated parasites (Figures 8B3 and 8C3) compared to discrete spherical apicoplasts found in daughter merozoites in untreated controls (Figures 8A1 and 8A3). This pattern of diffuse apicoplast fluorescence could also be a result of defective import of apicoplast proteins as has been reported previously with delayed death drugs [47].

It is also possible that curcumin does not enter the host erythrocyte at high concentrations. Hence, maximal exposure to the drug occurs in the very small time frame between the egress of merozoites from infected erythrocytes and invasion of new erythrocytes. It is only at this brief window of time that the extracellular merozoites are in contact with the drug present in the media. This is supported by the data showing nanomolar concentrations of curcumin in the intracellular compartment during the first and second cycles of parasites in culture, while extracellular concentrations of curcumin are approximately 1000 fold higher. If schizonts are shielded from curcumin in their intracellular niche and only exposed to the drug prior to the start of the second cycle when they egress, a delayed effect of curcumin would be expected. This is also consistent with the data showing that pre-treatment of erythrocytes with curcumin and presumably loading them with the drug does not affect parasite growth.

The points discussed above explore the possibility of curcumin having a pronounced effect on microtubule structures in the second cycle due to mechanisms unrelated to curcumin directly binding microtubules. Instead, if curcumin acts directly on tubulin, yet another explanation lies in the way antimitotic drugs exert their effect on microtubules. It has been noticed that at high concentrations of destabilizing drugs like vinblastine, there is inhibition of GTPase activity, preventing polymerization. However at much lower but pharmacologically significant concentrations, they affect the dynamic instability of the microtubules [56], [57]. Considering that *P. falciparum* tubulins have not been fluorescently tagged *in vivo* as yet, it is not possible to perform live cell microscopy to assess the effect of the drug on the dynamic properties of parasite microtubules. Nevertheless diffused micro-punctate structures observed in curcumin treated fixed parasites (Figure 6 B, C) might represent accumulated unpolymerized tubulin. This would be similar to observations with other microtubule disrupting drugs tested in *P. falciparum* [27], [35], [58] illustrated in Figure 7A, and different from the thick rods of microtubules observed after paclitaxel treatment, a tubulin stabilizing agent [22], [23], [59], [60] and Figure 7B. Interestingly, the figures are suggestive of the fact that although spindle structures are affected, there seems to be no difference in the outcome as far as the division of the nucleus is concerned. Neatly segmented nuclei appeared in the schizont stages of curcumin-treated cultures. These observations suggest that curcumin might exert its inhibitory effect by promoting aberrant mitosis, like other microtubule disrupting drugs, and not a complete mitotic block promoted by stabilizing drugs like paclitaxel.

### Conclusion

Considering curcumin is known to have multiple targets in *P. falciparum*, it would be interesting to understand their individual roles and which are necessary for their antiparasitic effect. We have tried to explore the effect of curcumin on *P. falciparum* microtubules and plausible mechanisms leading to this effect. Our molecular docking data suggests that curcumin might bind at the interface of alpha-beta tubulin heterodimer leading to altered microtubule morphology, as shown by our immunofluorescence studies. This is supported by results from drug combination assays with an antagonistic interaction between curcumin and colchicine suggesting competition for the same binding site. Alternatively, it might still be possible that curcumin does not bind directly to tubulin. The microtubule destabilizing effect could also be a part of global cell damage or due to off target effects of curcumin. Curcumin is known to affect a significant number of kinases in mammalian cells. Similar studies have not been done in *P. falciparum*, but a similar action in the parasites might have an indirect effect on the polymerization of microtubules. Irrespective of the cause, the damaged microtubules hinder cellular functions including apicoplast morphology. These lead to reduced parasite viability and are, at least partially, responsible for the antiparasitic action of curcumin.
